# A New Strategy for Enhancing the Oral Bioavailability of Drugs with Poor Water-Solubility and Low Liposolubility Based on Phospholipid Complex and Supersaturated SEDDS

**DOI:** 10.1371/journal.pone.0084530

**Published:** 2013-12-31

**Authors:** Hui Zhou, Jiangling Wan, Lei Wu, Tao Yi, Wei Liu, Huibi Xu, Xiangliang Yang

**Affiliations:** 1 National Engineering Research Center for Nanomedicine, College of Life Science and Technology, Huazhong University of Science and Technology, Wuhan, China; 2 State Key Laboratory of Quality Research in Chinese Medicine, Macau Institute for Applied Research in Medicine and Health, Macau University of Science and Technology, Taipa, Macau; Aristotle University of Thessaloniki, Greece

## Abstract

A novel supersaturated self-emulsifying drug delivery system (Super-SEDDS) loaded with scutellarin-phospholipid complex (SPC) was developed. The system aimed to address the limitations presented by conventional SEDDS as delivery carriers for drugs with poor water-solubility, low liposolubility and high dose. As an intermediate, SPC was first prepared based on the response surface design. The presence of amorphous scutellarin was demonstrated through differential scanning calorimetry (DSC) and X-ray diffraction (XRD), while enhanced liposolubility was confirmed through comparison with scutellarin powder via an octanol/water distribution test. On the basis of the solubility study and ternary phase diagram, Super-SEDDS containing SPC of up to 200% equilibrium solubility (S_eq_) was designed, which composed of ethyl oleate, Cremophor RH40 and Transcutol HP with a ratio of 60∶25∶15 (w/w%). The subsequent *in vitro* lipolysis study and *ex vivo* intestinal absorption test indicated that Super-SEDDS enhanced the cumulative dissolution from 70% to 100% and improved the intestinal absorption from 0.04 to 0.12 µg/cm^2^ compared with scutellarin powder. Furthermore, an *in vivo* study demonstrated that Super-SEDDS achieved the AUC_0-t_ of scutellarin up to approximate 1.7-fold as scutellarin powder. It was also proved superior to SPC and the conventional SEDDS. Super-SEDDS showed great potential for expanding the usage of SEDDS and could act as an alternative to conventional SEDDS.

## Introduction

Self-emulsifying drug delivery systems (SEDDS), the isotropic mixtures of oils, surfactants and co-surfactants, have been shown to successfully enhance the oral bioavailability of several compounds with low solubility but high permeability [1], [2], [3]. These compounds would normally be classified in the bio-pharmaceutics classification system (BCS) class II [4]. Compared with other drug delivery systems, in particular nano-crystal, SEDDS present superiority by encapsulating BCS class II compounds within the hydrophobic core in a dissolved state [5]. While traditionally, two principles need to be obeyed when using SEDDS as drug delivery carriers. Firstly, high lipophilic drugs are suggested, in order to be abidingly dissolved in the SEDDS pre-concentrate even at high dose [6]. Secondly, low drug loading rates are recommended as to prevent drugs from precipitation after SEDDS are emulsified in the gastrointestinal (GI) tract [7]. Therefore, compounds classified as BCS class IV with low solubility and poor permeability, as well as some extracts generated from medicinal plants, are restricted for use with SEDDS if administrated at high dose [8].

In order to overcome these limitations, two major strategies are suggested as follows. Firstly, phospholipid complex method, which has frequently been used to modify compounds, could be applied to reverse the poor liposolubility of various compounds to match the delivery style of SEDDS [9], [10], [11]. For example, matrine phospholipid complex was found superior to matrine powder, as it had enhanced solubility in octanol but reduced in water [9]. This anticipated the inversion of the octanol/water distribution. A similar effect was seen when morin was prepared as phospholipid complex [12]. The second method, known as Super-SEDDS, has received a considerable amount of attention for high-dose administrations of drugs where conventional SEDDS has proven inept. In the early stages, Super-SEDDS was designed for preventing the precipitation of drugs through the addition of precipitation inhibitors such as polyvinyl pyrrolidone (PVP) and hydroxypropyl methylcellulose (HPMC) [13], [14]. Benefiting from this, a supersaturated solution of drugs might be formed and temporarily maintained after SEDDS is emulsified in the GI tract, which could facilitate the absorption of drugs. However, the drug loading rate still has not been obviously enhanced with this method. Although the original results seem discouraging, more recent findings obtained from *in vitro* lipolysis revealed that the amorphous precipitation of a drug was sometimes insignificant for absorption [15]. Thomas et al. prepared Super-SEDDS with longer ultra-sonication and a warmer temperature, loading with halofantrine at a dose of 150% S_eq_ [16]. The precipitates of halofantrine obtained from *in vitro* lipolysis of Super-SEDDS were confirmed amorphous by X-ray powder diffraction (XRPD). In addition, a single capsule of Super-SEDDS achieved similar AUC and C_max_ results as seen from two capsules of conventional SEDDS. This strategy can thus be regarded as an important approach with high potential applications. However, many compounds might still be difficult for delivery through the Super-SEDDS method, as their precipitates cannot be efficiently absorbed.

In this study, phospholipid complex and Super-SEDDS were combined for the first time to expand the usage of SEDDS. The preparation of SPC was optimized by surface response design and its physicochemical properties, including physical state and octanol-water distribution, were investigated. Based on a solubility study and ternary phase diagram, Super-SEDDS containing SPC up to 200% of S_eq_ were prepared, followed by an *in vitro* lipolysis study and additional *ex vivo* intestinal absorption research. Finally, *in vivo* study was performed on rats to detect the oral absorption of scutellarin dosing in Super-SEDDS.

**Figure 1:**
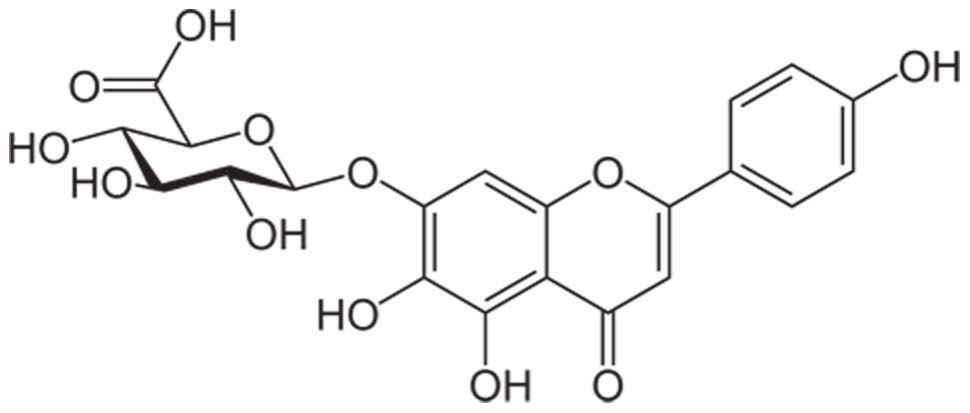
The structure of scutellarin.

Scutellarin (Figure 1), the main component of breviscapine extracted from chinese herb *Erigerin breviscapus* (Vant.) Hand-Mazz [17], was taken as a model compound with low water solubility (14.4±0.7 µg/mL), poor membrane permeability (Log *P* = − 2.56±0.04) [18], but high oral dose (120∼420 mg per day). It is widely used in the treatment of cardiovascular or cerebrovascular diseases in China. In our previous work, scutellarin phospholipid complex (SPC) was prepared to achieve enhanced *ex vivo* intestinal absorption, as compared with scutellarin powder [19]. Similarly, SEDDS were tried to promote solubilization of scutellarin in several other studies [20], [21]. Unfortunately, these studies stalled on the *in vitro* and *ex vivo* behavior of SEDDS, but did not provide any *in vivo* results.

## Materials and Methods

### Ethics Statement

All surgical and experimental procedures were supervised and approved by the Animal Experimentation Ethics Committee of Huazhong University of Science and Technology.

### Materials

Scutellarin (92.7% purity) was purchased from Yunnan Phytopharmaceutical Co., Ltd. (Kunming, China). Scutellarin standard (purity >98%, as determined by HPLC) and rutin standard (internal standard, purity >98%) were obtained from the National Institutes for Food and Drug Control (Beijing, China). Soybean phospholipid (Lipoid S75) was supplied by Shanghai Toshisun Biology and Technology Co., Ltd. (Shanghai, China). Ethyl oleate and Tween 80 were acquired from China National Pharmaceutical Group Corporation (Shanghai, China). Maisine 35-1, Labrafac Lipophile WL 1349, Capryol 90, Transcutol HP and Labrasol were obtained from Gattefossé (Saint-Priest Cedex, France). Cremophor RH40 and Cremophor EL were provided by BASF (Ludwigshafen, Germany). Bile salt, trizma® maleate and pancreatic lipase were obtained from Sigma Chemical Company (Shanghai, China). HPLC grade acetonitrile and methanol were purchased from Merck (Darmstadt, Germany). Purified water was prepared by a Millipore Milli-Q ultrapure water purification system (Billerica, MA, USA). Male Sprague-Dawley rats (weighing 300∼350 g) were purchased from the Tongji Medical College of Huazhong University of Science and Technology (Wuhan, China). All other chemicals used were of analytical grade.

### Preparation of SPC

Scutellarin-phospholipid complex was prepared by a solvent evaporation method. Initially, scutellarin powder and soybean phospholipid were dispersed in alcohol at a pre-designed ratio (w/w). The dispersion was subsequently gently stirred in a warm water bath (45∼55°C) and the formed solution was then continually heated (45∼55°C) with the use of a rotary evaporator to evaporate alcohol. The obtained SPC was further dried overnight in an oven at room temperature and stored in a desiccator until use.

The optimization of the preparation process was carried out through a three-factor, three-level central composite design. According to preliminary studies, three factors including the ratio of scutellarin to phospholipid (X1), the temperature of the water bath (X2) and the drug concentration in solvent (X3), were regarded as independent variables with the highest influence on combination percentage (Y). Favorable levels of the three independent variables were defined as shown in Table 1. The combination percentage was calculated using the following equation: 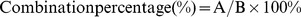
Where, A was the amount of combined scutellarin in SPC, and B was the mass of scutellarin initially added. The mass of combined scutellarin was measured after SPC was dissolved by chloroform, in which scutellarin powder was found insoluble. The whole process of response surface design including experiences arrangement, equation fitting and condition optimization was performed by Design-Expert® v8.0.6 software (Stat-Ease Inc., Minneapolis, USA).

**Table 1:** Independent variables and their correspondent values for the optimization of SPC preparation, using the central composite design.

Independent variables	Levels used		
	Low	Mean	High
X1 = Scutellarin/Phospholipid (w/w)	0.50	0.75	1.00
X2 = Temperature (°C)	45	50	55
X3 = Concentration (mg/mL)	5	10	15

### Differential Scanning Calorimetry of SPC

In order to detect thermodynamic properties, a differential scanning calorimeter (Diamond DSC, PerkinElmer Instruments, USA) was employed to take thermograms of scutellarin powder, soybean phospholipid, physical mixture and SPC. The samples were sealed in an aluminum crimp cell and heated from 50°C to 300°C at a rate of 10°C/min.

### X-ray Diffraction of SPC

The physical states of scutellarin powder, soybean phospholipid, physical mixture and SPC were investigated by using an X-ray diffractometer (Bruker D8 advance, Bruker, Germany) at room temperature with a voltage of 40 kV and a current of 40 mA. All samples were scanned over a range of 2 θ angles from 3° to 65° with an angular increment of 0.02° per second.

### Octanol-water Distribution Coefficient of SPC

The octanol-water distribution coefficients of SPC and scutellarin powder were determined by using balanced solvent systems, which were composed of octanol and buffer solution. Briefly, octanol saturated buffer solutions (gradient pH 2∼8) and buffer saturated octanol were prepared, followed by dispersing appropriate amount of SPC or scutellarin powder into the buffer saturated octanol. Then, the octanol saturated buffer solutions and the drug added octanol solutions were mixed at a ratio of 1∶1 (v/v) and shaken for 4 h to achieve equilibrium. After 12 h balance in a separatory funnel, the mixture was phase separated and samples were drawed out from both octanol and buffer phase for HPLC detection (as described later). The concentrations of scutellarin were labeled as C_o_ (octanol) and C_S_ (buffer solution), respectively, and then Log *D* (distribution coefficient) was calculated.

### Solubility Study of SPC

A solubility study was initiated by adding a surfeit of SPC to approximate 2 g excipients. The initial suspensions were vigorously shaken for 2 h by vortex shaker (Vortex 3, IKA, Staufen, Germany), followed by continually shaking in a constant-temperature shaker at 37°C for 48 h to reach equilibrium according to the preliminary experience. The final suspensions were centrifuged at a speed of 10,000 rpm for 10 min at room temperature (Centrifuge 5810R, Eppendorf, Germany), and the supernatant was appropriately diluted with methanol for the quantification analysis of scutellarin by HPLC method (as described later). As a control, a solubility study of scutellarin powder was also carried out with the same method.

### Construction of the Ternary Phase Diagram

Pre-concentrate was composed of ethyl oleate, Transcutol HP and surfactant (Tween 80 or Cremophor RH40), in which the amount of surfactants was fixed at predetermined levels (12.5, 25.0, 37.5, 50.0, 62.5, 75.0, 87.5 w/w %), and the ratio of ethyl oleate to Transcutol HP was simultaneously regulated from 1∶9 to 9∶1. After mixing was completed, approximate 100 mg of pre-concentrate was dispersed in 5 mL of phosphate buffer solution (PBS, pH 6.8) through gentle agitation at 37°C. Then, the transmittance of dispersion was measured at 600 nm, setting the transmittance of PBS as 100%. The dispersions with a transmittance value higher than 10% were considered as successfully self-emulsifying. Consequently, the emulsifying region was identified for both the Tween 80 system and Cremophor RH40 system.

### Determination of the Super-SEDDS Formulation

According to the obtained ternary phase diagrams, emulsifiable formulations composed of SPC saturated oil, surfactant and co-surfactant were diluted by PBS at a ratio of 1∶200 (w/v). The optimal SEDDS formulation was selected according to droplet size and polydispersity index (PDI) as described next section, as well as drug concentration of emulsion after 2 h deposition. The equilibrium solubility of SPC in the selected SEDDS was measured in accordance with above mentioned. Finally, Super-SEDDS loaded with SPC equivalent to 200% of S_eq_ was prepared for further study, and conventional SEDDS with 50% of S_eq_ was selected as the control sample.

### Morphology of Super-SEDDS

The conventional SEDDS and Super-SEDDS were emulsified by PBS at a dilution of 200 (w/v). The droplet size and zeta-potential of emulsion were measured by dynamic light scattering (DLS) and phase analysis light scattering (PALS), using Malvern Zetasizer Nano (Malvern Instruments, Worcestershire, UK). The detection was carried out by scattered light with a 90°C angle at the temperature of 25°C.

A drop of the emulsion was placed on a carbon-coated copper grid. It adhered for 15 min and was then absorbed carefully by the filter papers. The remaining film on the grid was negatively stained by 2% (w/v) phosphor tungstic acid (pH 7.0) within 2 min. All the copper grids were kept dry in a desiccator until observed on a transmission electron microscope (H-7000FA, Hitachi Ltd., Japan).

### *In Vitro* Lipolysis Study

*In vitro* lipolysis studies were performed as previously described [16], [22] with minor modifications. Briefly, lipolysis medium was prepared in advance, containing 5 mM bile salt, 2 mM trizma maleate buffer, 1.25 mM phosphatidyl choline, and 150 mM sodium chloride, and was adjusted to pH 6.5 with 0.1 M NaOH. Then according to the single-dosage of commercial oral solution detailed as 70 mg scutellarin administrated into 900 mL GI medium, scutellarin powder, SPC, conventional SEDDS and super-SEDDS with a dose of 20 mg (equivalent to scutellarin) were dispersed in 250 mL warmed lipolysis medium (37°C), with a stirring rate of 100 rpm. After that, lipolysis was initiated by adding 50 mL freshly prepared pancreatic lipase solution (2000 TBU/mL, pH 6.5, 37°C), then controlled by constantly pumping 0.15 M CaCl_2_ with a rate of 0.25 mL/min. Over the lipolysis period, pH value was maintained at 6.5 by a pH-stat device (902 Titrando, Metrohm, Switzerland). At pre-determined time intervals (5, 10, 20, 30, 45, 60, 90, 120 min), 5 mL digested medium was taken and filtered by 0.45 µm polypropylene membrane. The filtered samples were immediately diluted 10 times with acetonitrile to stop lipolysis and induce protein precipitation. At last, the diluted samples were centrifuged at 10,000 rpm for 10 min (Centrifuge 5810R, Eppendorf, Germany), and subsequently analyzed by HPLC (as mentioned later).

### *Ex Vivo* Intestinal Absorption Study

An everted intestinal sac experiment, modified according to the method previously described [23], was adopted to evaluate the *ex vivo* intestinal absorption of scutellarin. Kreb-Ringer’s (K–R) solution, containing 342.0 mM NaCl, 6.7 mM KCl, 5.9 mM CaCl_2_·2H_2_O, 5.3 mM MgCl_2_, 59.5 mM NaHCO_3_, 2.1 mM NaH_2_PO_4_ and 5.5 mM glucose, was prepared in advance. Half was cooled in 4°C refrigerator and the rest was warmed in a 37°C water bath. Scutellarin powder, SPC, conventional SEDDS and Super-SEDDS were dispersed in warmed K–R solution with a concentration of 50 µg/mL scutellarin, approximate to the lowest released concentration of drug during the lipolysis test. Male Sprague-Dawley (SD) rats were fasted overnight and then anesthetized with an intraperitoneal injection of chloral hydrated (10%, w/v) at a dose of 0.4 mL per 100 g. After dissection of the abdominal wall, the jejunum (21 cm from the pylorus) was carefully isolated without any mesentery, clipped into segments of 10 cm, and quickly rinsed in cooled K–R solution. The segments were then tied securely with cotton thread at one end, everted by a smooth glass rod, rinsed to clean the mucus, plugged in with a plastic tube at the other end, over brim injected with 1∼2 mL drug-free K–R solution and suspended in a 40 mL test K–R solution at 37°C with O_2_/CO_2_ (95%/5%) aerating. At each predetermined time interval (0, 15, 30, 45, 60, 90, 120, 150 and 180 min), 0.1 mL of the intracapsular sample was taken and the same volume of warmed fresh K–R solution was added. The samples were centrifuged at a speed of 10,000 rpm for 10 min before HPLC analysis. After the tests, the length and width of the intestinal segments were measured for the calculation of uptake amount per unit area.

### *In Vivo* Study

20 male SD rats (300∼350 g) were fasted but allowed free access to water for 12 h, then divided randomly into 4 groups for the pharmacokinetics experiments of scutellarin powder, SPC, conventional SEDDS and Super-SEDDS. In order to avoid failure detection of plasma drug, the various formulations were orally administrated to rats at a dose of 40 mg/kg, according to the highest dosage of commercial oral solution, with a ceiling volume of 10 mL/kg when dispersed by purified water. The blood samples of 0.5 mL were collected in a 1.5 mL heparin treated EP tube from the orbital venous prior to the dose and at 0.5, 1, 2, 3, 4, 5, 6, 8, 10, 12, 24 and 36 h after the dose. The plasma was immediately isolated from whole blood by centrifugation (4000 rpm, 10 min) at room temperature and then stored in a −80°C freezer until analysis.

### Quantitative Analysis of Plasma Samples

An HPLC analysis system (HP 1100 series, Agilent Technologies, Wilmington, Germany) of a quaternary pump, an autosampler and a UV detector with an ODS-C18 column (250 mm×4.6 mm i.d., Sepax Technologies Inc., Delaware, USA), was used for the determination of scutellarin. The mobile phase was a mixture of methanol, acetonitrile and 50 mM KH_2_PO_4_, at a ratio of 22∶15:63 (v/v/v), with pH of 2.5 adjusted by phosphoric acid. The detection was carried out at a wavelength of 335 nm, with a flow rate of 1.0 mL/min at room temperature.

200 µL of thawed plasma was removed to a 10 mL EP tube. 50 µL of internal standard working solution (20 µg/mL of rutin in methanol) was added and subsequently acidified by 100 µL of phosphoric acid (10 wt %) for 10 min. Then, 3 mL of ethyl acetate was infused for extracting scutellarin from plasma. After 30 min vortex, samples were centrifuged at 4000 rpm for 10 min, and the isolated supernatant was concentrated under nitrogen gas flow in bath of 40°C. Before HPLC detection, it needed to be re-dissolved by 100 µL of HPLC mobile phase and centrifuged at 10,000 rpm for 5 min. The whole analytical methodology including plasma treatment and HPLC detection was validated by accuracy and precision (see Table 2) as previously described [24].

**Table 2:** The validation of analytical methodology including accuracy and precision from quality control samples of rat plasma extracts (n = 3 days, five replicates per day).

Added concentration (ng/mL)	Found concentration (ng/mL)	Accuracy (%)	Precision (%)	
			Intra-run	Inter-run
125	122	97.6	5.3	6.2
1250	1245	99.6	7.8	7.7
12500	12100	96.8	9.7	6.4

### Statistical Analysis

The plasma data was analyzed in batches by DAS 2.0 (Mathematical Pharmacology Professional Committee of China, Shanghai, China). Peak plasma concentration (C_max_) and the time to reach C_max_ (T_max_) were based on measured values. Area under the curve (AUC_0-t_) was calculated via a trapezoidal area method. Half-life period (T_1/2z_) was simulated by statistical moment method. Herein, T_1/2z_ = 0.693/Zeta, where Zeta is the terminal slope of c-t curve (originating from the last 5 points of curve). All values were expressed as the mean ± standard deviation. The significant differences between each group were determined by one way analysis of variance (ANOVA) using the statistical package for social sciences software (SPSS® version 17.0, SPSS Inc., Shanghai, China), with the significance defined as *p*<0.05 and high significance as *p*<0.001.

## Results and Discussion

### Preparation of SPC

Response surface methodology (RSM), a practical modeling tool consisting of mathematical and statistical techniques, is usually employed for predicting the effects of independent variables on dependent variables separately and interactively [25]. Table 3 presents the experimental data obtained on different conditions arranged by Design-Expert® based on the preliminary study. The fitted model was established from the equation: 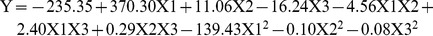


**Table 3:** Different combinations of independent variables contributing to their response values (n = 3).

Run	X1 (w/w)	X2 (°C)	X3 (mg/mL)	Y (%)
1	0.50	55.0	15.00	86.4±0.9
2	1.00	55.0	15.00	59.7±0.8
3	0.33	50.0	10.00	83.3±0.6
4	1.00	45.0	5.00	81.9±1.8
5	0.75	50.0	10.00	83.6±1.3
6	1.17	50.0	10.00	45.8±1.1
7	0.75	50.0	18.41	77.2±1.4
8	0.75	50.0	10.00	90.0±0.7
9	1.00	45.0	15.00	51.7±1.1
10	1.00	55.0	5.00	61.7±0.7
11	0.75	41.6	10.00	76.6±0.5
12	0.75	50.0	10.00	90.7±0.8
13	0.75	58.4	10.00	87.8±1.1
14	0.50	45.0	5.00	97.8±1.8
15	0.50	45.0	15.00	55.2±0.8
16	0.50	55.0	5.00	100.0±1.2
17	0.75	50.0	1.59	93.8±1.5

**Figure 2:**
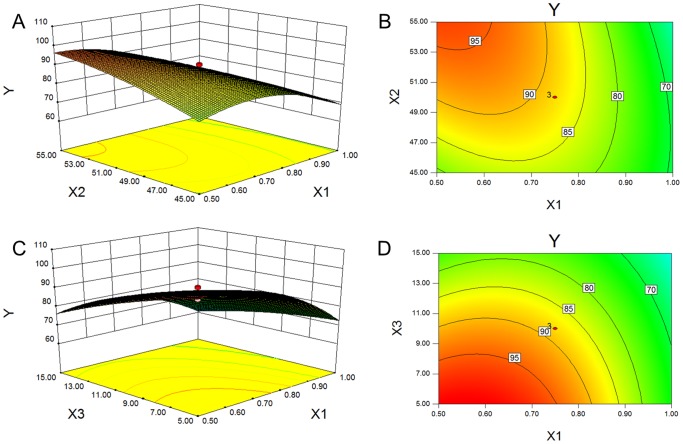
Response surface plots. The significant (*p*<0.05) interaction effects for combination percentage, as a function of X1/X2 and X1/X3, are presented as 3D surfaces (A, C) and contours (B, D) respectively. X1: the ratio of scutellarin to phospholipid; X2: the temperature of the water bath; X3: the drug concentration in solvent.

According to this equation, the effects of independent variables (X1, X2 and X3) and their interactions on dependent variable (Y) could be quantitated. The fitness of the model and independent variables were evaluated using F-value and *p*-value. As Table 4 shows, the model was well-matched (*p*<0.05), and the model terms X1, X2, X3, X1X2, X2X3 and X1^2^ were significant (*p*<0.05). Surface response design proved advantageous as the optimized level of each independent variable could be presented directly via a two-dimension or three-dimension graph. Figure 2 shows the response surface plot illustrating the significant (*p*<0.05) interaction effect of independent variables on combination percentage. The combination percentage evidently rose as the ratio of scutellarin to phospholipid approached 0.5 (w/w), the temperature increased to 55°C and the drug concentration decreased to 5.0 mg/mL. The combination of scutellarin and phospholipid responded based on electrostatic interaction, suggesting that a complete combination only occurred if the ratio of scutellarin to phospholipid was 0.5 in mass (equivalent to 1∶1 in molar). The higher temperature of solvent was shown to promote molecular movement, which facilitated the conjugation of molecules. However, excessive temperatures are normally discouraged as they can promote the degradation of phospholipid [26]. A lower drug concentration in solvent was suggested to bring a higher percentage combination, but this resulted in a waste of solvent. Finally, considering both of the optimized solution and practical condition, the parameters of X1, X2 and X3 were adjusted to 0.5, 60°C and 10.0 mg/mL, with the combination percentage of 97.7±2.3%.

**Table 4:** Analysis of variance (ANOVA) of regression coefficients using F-value and *p*-value.

Source	F-value	*p*-value
Model	27.8355	0.0001*
X1	90.38592	<0.0001*
X2	6.693164	0.0361*
X3	62.72153	<0.0001*
X1X2	14.78878	0.0063*
X1X3	4.070043	0.0834
X2X3	23.05659	0.0020*
X1^2^	48.57951	0.0002*
X2^2^	3.91389	0.0884
X3^2^	2.583786	0.1520

Where, Xi, XiXj and Xi^2^ are the linear and interaction terms of the quadratic polynomial equation, respectively. The significant (*p*<0.05) terms are labeled with *.

### Differential Scanning Calorimetry

**Figure 3:**
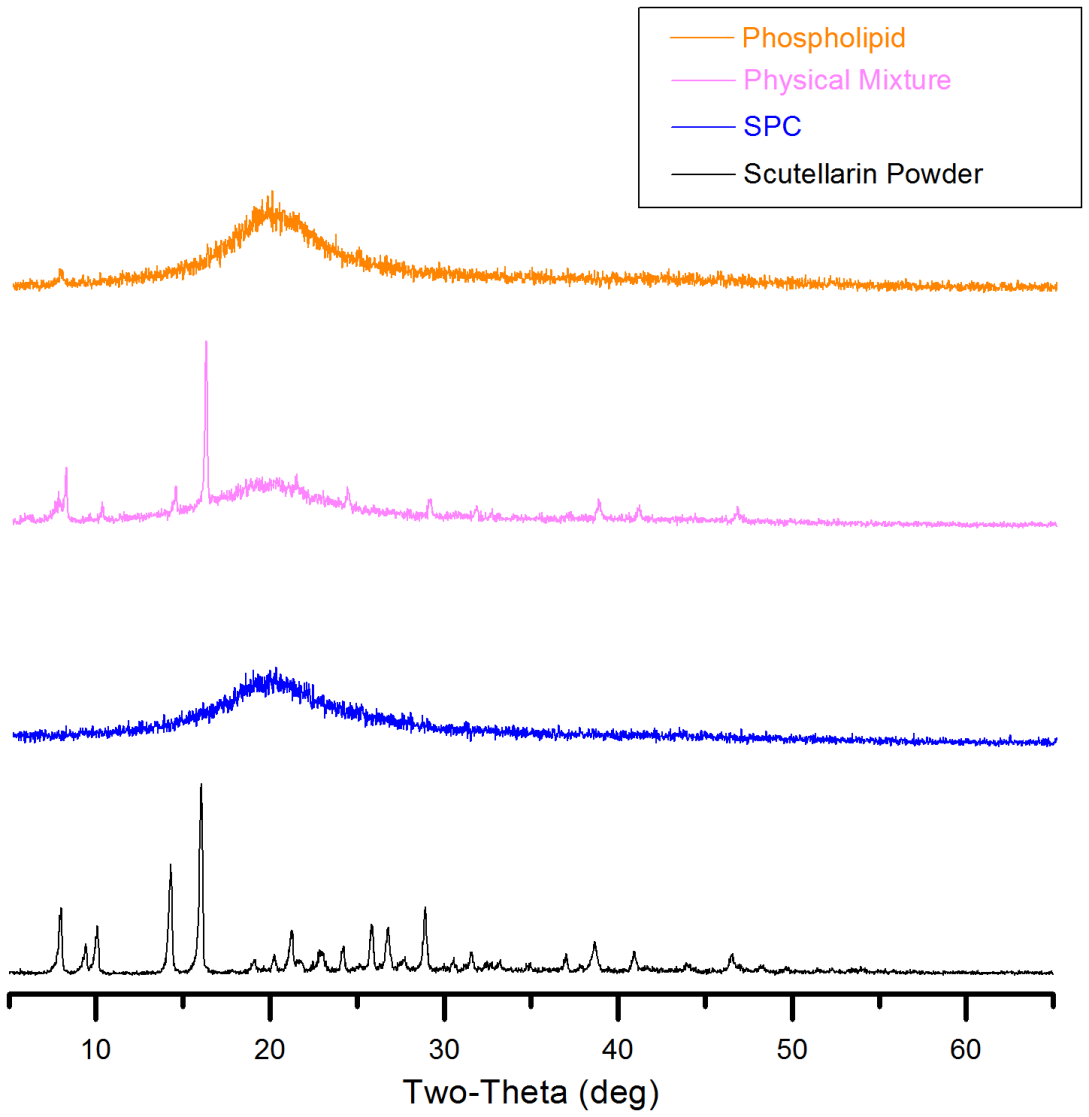
DSC thermograms of phospholipid, physical mixture, SPC and scutellarin powder. All samples were heated from 50°C to 300°C at a rate of 10°C/min.

DSC was applied to confirm the alteration of physical state of scutellarin contained in the SPC. The DSC curves of scutellarin powder, phospholipid, physical mixture and SPC are shown in Figure 3. Scutellarin powder showed an endothermal peak at 182°C, revealing the phase transition of crystal scutellarin. Meanwhile, the phospholipid began to thermally decompose when the temperature rose over 200°C. Evidently, the physical mixture displayed both endothermal peaks at 180°C (slightly lower than that of scutellarin powder) and thermal decomposition over 200°C (similar to phospholipid). The drift of endothermal peak might have resulted from the interaction between melted phospholipid and scutellarin powder, which might have promoted a phase transition. However, both endothermal peak and thermal decomposition disappeared in the curve of SPC, indicating the amorphous state of scutellarin presented in the SPC, as a function of the combination of polarity terminals between scutellarin and phospholipid. In other words, the amorphous state of scuttellarin demonstrated the successful preparation of SPC.

### X-ray Diffraction

**Figure 4:**
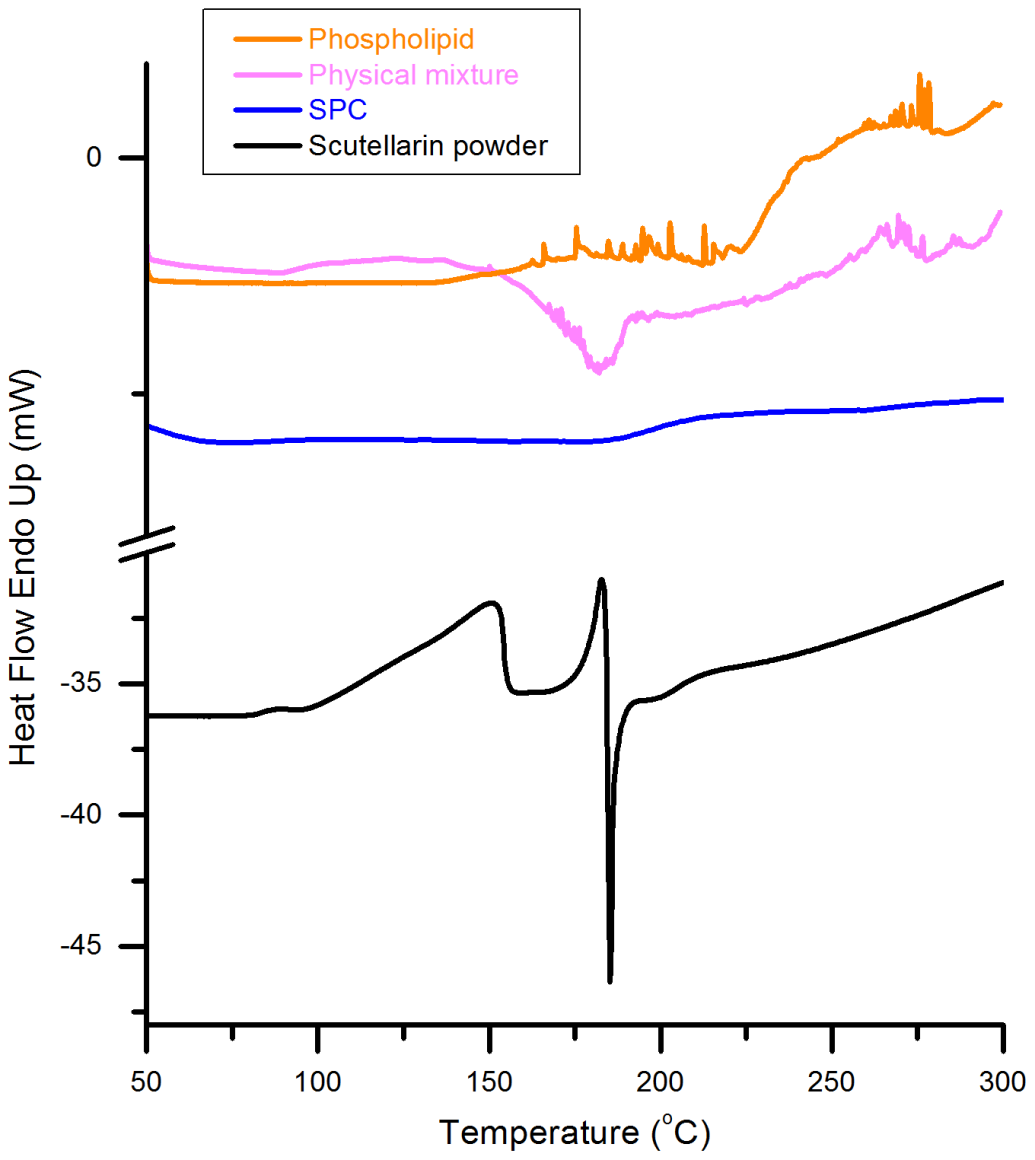
X-ray diffraction patterns of phospholipid, physical mixture, SPC and scutellarin powder. All samples were scanned over a range of 2 θ angles from 3°C to 65°C with an angular increment of 0.02°C per second.

In order to further investigate the physical state of scutellarin in SPC, XRD experiments were performed. The XRD patterns of scutellarin powder, phospholipid, physical mixture and SPC are presented in Figure 4. There was an abundance of peaks, indicating a crystal microstructure in scutellarin powder. This would suggest that the mixture pattern maintained the contour of phospholipid pattern, with the addition of partial characteristic peaks of scutellarin powder. In contrast, SPC dropped the crystal property of scutellarin powder completely, which could be attributed to the electrostatic interaction between scutellarin powder and phospholipid.

### Octanol-water Distribution Coefficient

**Figure 5:**
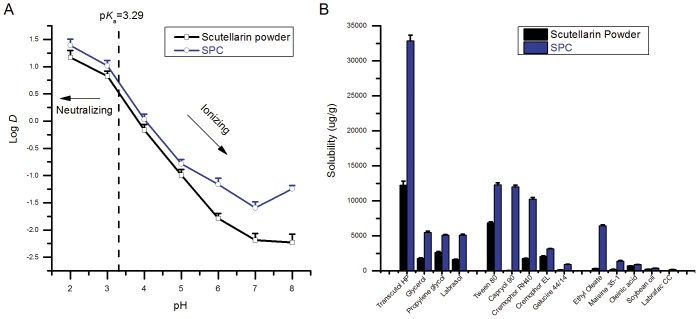
Lipophilicify evaluation and solubility study of scutellarin powder and SPC. Octanol-water distribution coefficients of scutellarin powder (black line) and SPC (blue line) were measured in the range of pH 2∼8 (A). Solubilities of scutellarin powder (black column) and SPC (blue column) in different vehicles were compared. Each test was performed 3 times in parallel (B).

Octanol-water distribution coefficient (Log *D*), which is usually regarded as a reflection of permeability, can determine the fate of a compound in GI tract. The Log *D* values of scutellarin and SPC corresponding to different pH values are presented in Figure 5A. As it shows, the Log *D* values of SPC were higher than those of scutellarin in the range of pH 2∼8, particularly at pH 6∼8, which revealing a conversion of ionization and distribution behavior of scutellarin in both octanol and buffer. As the p*K*_a_ of scutellarin is 3.29 [27], it could be concluded that majority of scutellarin will be neutral if pH <3.29, in contrast, ionic if pH >3.29. For SPC, the participation of phospholipid might cause slight drift of p*K*_a_ value, and perhaps benefit higher distribution of drug in octanol no matter on acidic or basic condition.

### Solubility of SPC

The solubility of drugs in SEDDS was mainly based on solubility in various compositions, including oils, surfactants and co-surfactants. It was sometimes much higher due to the synergistic effect. The solubilities of scutellarin powder and SPC in various vehicles are compared in Figure 5B. It was evident that the solubility of scutellarin in most vehicles was highly enhanced by SPC. Among the oils tested, ethyl oleate was selected as the best candidate, as it has an outstanding solubility of SPC. For the same reason, Transcutol HP was selected as the only co-surfactant. Compared with oils, surfactants provide relatively higher solubility of SPC, in which the top three are Tween 80, Capryol 90 and Cremophor RH40. Tween 80 is commonly used as a surfactant in the formulations of emulsion or microemulsion, with a HLB value of 15. Cremophor RH40 is an excellent surfactant for o/w emulsion system, depending on its suitable HLB value and strong emulsifying capacity. However, Capryol 90 is a water insoluble surfactant, which is incompetent as an emulsifier used in o/w emulsion and is innately incompatible with ethyl oleate and Transcutol HP. Therefore, only Tween 80 and Cremophor RH40 were selected as the candidates for further ternary phase diagram studies.

### Construction of Ternary Phase Diagram

**Figure 6:**
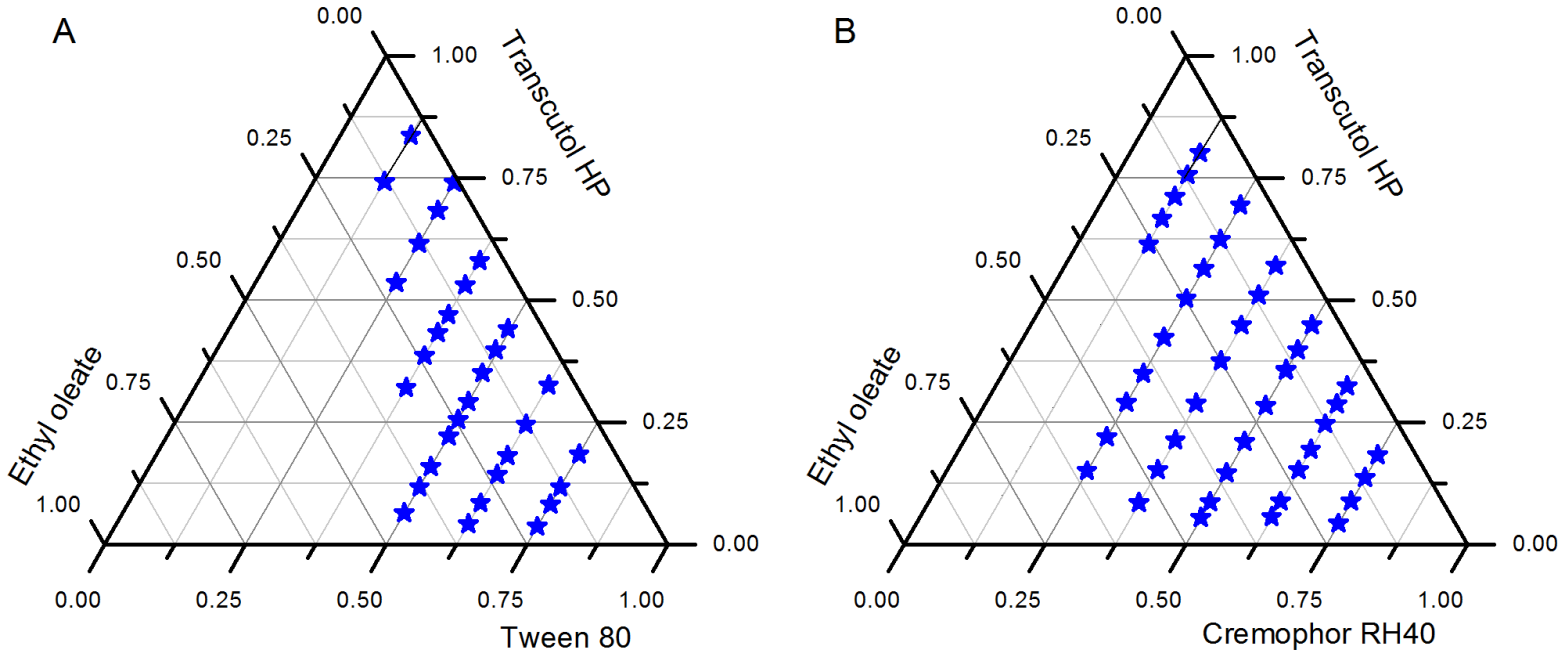
Ternary phase diagrams. A system composed of ethyl oleate, Tween 80 and Transcutol HP (A); Another system composed of ethyl oleate, Cremophor RH40 and Transcutol HP (B); Microemulsifying area was assigned with asterisks.

A ternary phase diagram is usually adopted as guidance for the formulation of oil, surfactant and cosurfactant during the development of an emulsion. The ternary phase diagrams of Tween 80 and Cremophor RH40 were constructed as seen in Figure 6. The emulsion region of Cremophor RH40 can be seen to be much larger than that of Tween 80, which would suggest that Cremophor RH40 has a stronger emulsifying capability and should be selected as the only surfactant.

### Determination of Super-SEDDS

It is sure that surfactants used in SEDDS have cytotoxicity [28], including Cremophor RH40 [29]. For this reason, the amount of Cremophor RH40 was controlled between 20∼50%. At the same time, ethyl oleate was controlled between 40∼60% and Transcutol HP was controlled between 5∼30%. The screening of SEDDS formulations was carried out based on size, PDI and drug loading capability of the emulsified droplet. Finally, the selected SEDDS was composed of 60% ethyl oleate, 25% Cremophor RH40 and 15% Transcutol HP, with a S_eq_ of 39.7±1.2 mg/g (equivalent to scutellarin). Super-SEDDS containing SPC up to 200% S_eq_ was prepared, and the loading rate of SPC was hard to be further enhanced due to the limited compatibility of SPC to SEDDS. Conventional SEDDS loaded with SPC at 50% S_eq_ were also prepared for control experiments. It would be certainly more rational to include SEDDS loaded directly with scutellarin as a control formulation, to help judge the additional benefit from the combining SPC with SEDDS. However, it was not possible due to the limited liposolubility of the drug.

**Figure 7:**
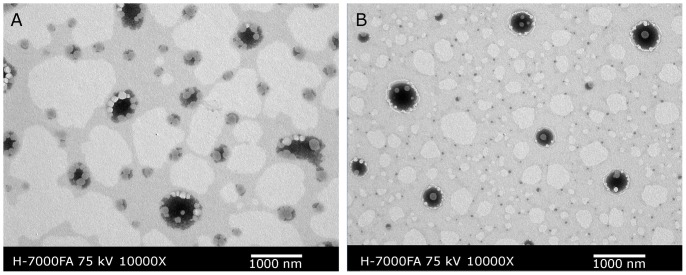
TEM graphs of conventional SEDDS (A) and Super-SEDDS (B).

As the DLS data indicated, the emulsion originating from Super-SEDDS was much smaller in size (386.0±10.4 nm vs. 759.5±32.5 nm) than that originating from conventional SEDDS, with narrower droplet size distribution (PDI 0.237±0.028 vs. 0.391±0.055) and more negative zeta-potential (−41.9±0.4 mV vs. −21.2±3.2 mV). This could be attributed to the innate emulsifying effect seen from phospholipid and the negative charges of phosphatidyl choline. Figure 7 shows the transmission electron microscopy (TEM) graphs of conventional SEDDS and Super-SEDDS. The use of abundant phospholipid in Super-SEDDS played an important role in the construction of emulsions, resulting in the avoidance of aggregation.

### *In Vitro* Lipolysis Study

**Figure 8:**
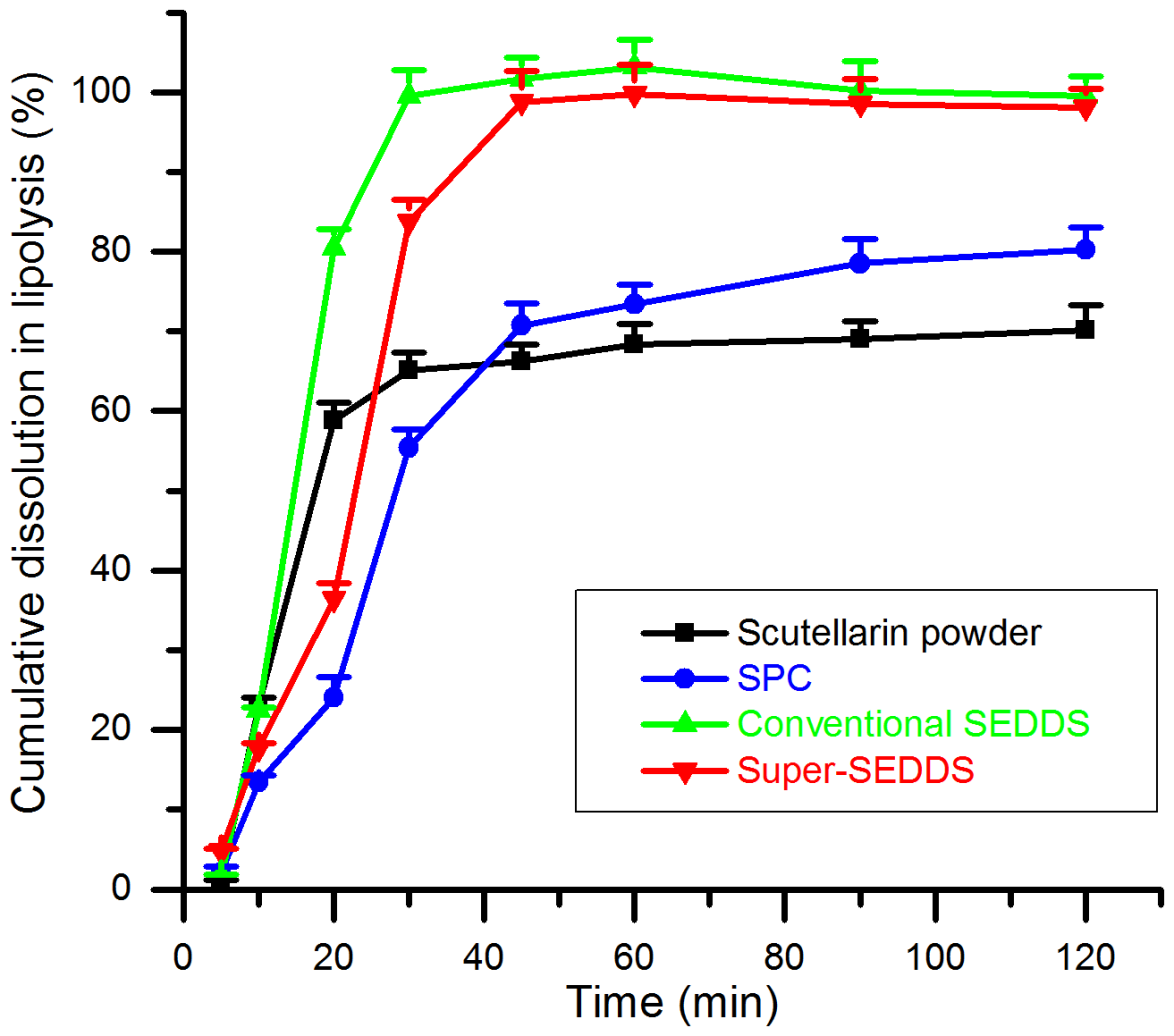
The *in vitro* lipolysis profiles. Lipolysis release properties of scutellarin from scutellarin powder (black), SPC (blue), conventional SEDDS (green) and Super-SEDDS (red), evaluated as cumulative dissolution percentages, were investigated in lipolysis medium (pH 6.5, with 2000 TBU/mL pancreatic lipase) at 37°C (n = 3).

The *in vitro* release profiles of scutellarin powder, SPC, conventional SEDDS and Super-SEDDS as illustrated in Figure 8, reflected the dissolution rate and solubilization efficiency of formulations after lipolysis. In the beginning, the drug release rate of SPC was the slowest, ascribing to the poor dispersibility of SPC. For the same reason, release rate from super-SEDDS was much slower than conventional SEDDS. However, the cumulative dissolution percentage of SPC was 80.2±2.8%, which was significantly higher than that of scutellarin powder (70.1±3.2%). This was thought to be due to the solubilization effect of phospholipid. The drug release rates and cumulative dissolution percentages of conventional SEDDS (99.5±2.5%) and Super-SEDDS (98.1±2.3%) were much higher than those of SPC, not to mention scutellarin powder. This could be attributed to the accelerated dissolution and enhanced solubility resulting from emulsifying and lipolysis. Surely, the digestible lipid in SEDDS would generate fatty acids during the lipolysis, which facilitates in the formation and rapid incorporation of drug into bile-salt-lipid mixed micelle with consequent increased solubility, in contrast, the absence of lipid in scutellarin powder would cause consequent precipitation.

### *Ex Vivo* Intestinal Absorption Study

**Figure 9:**
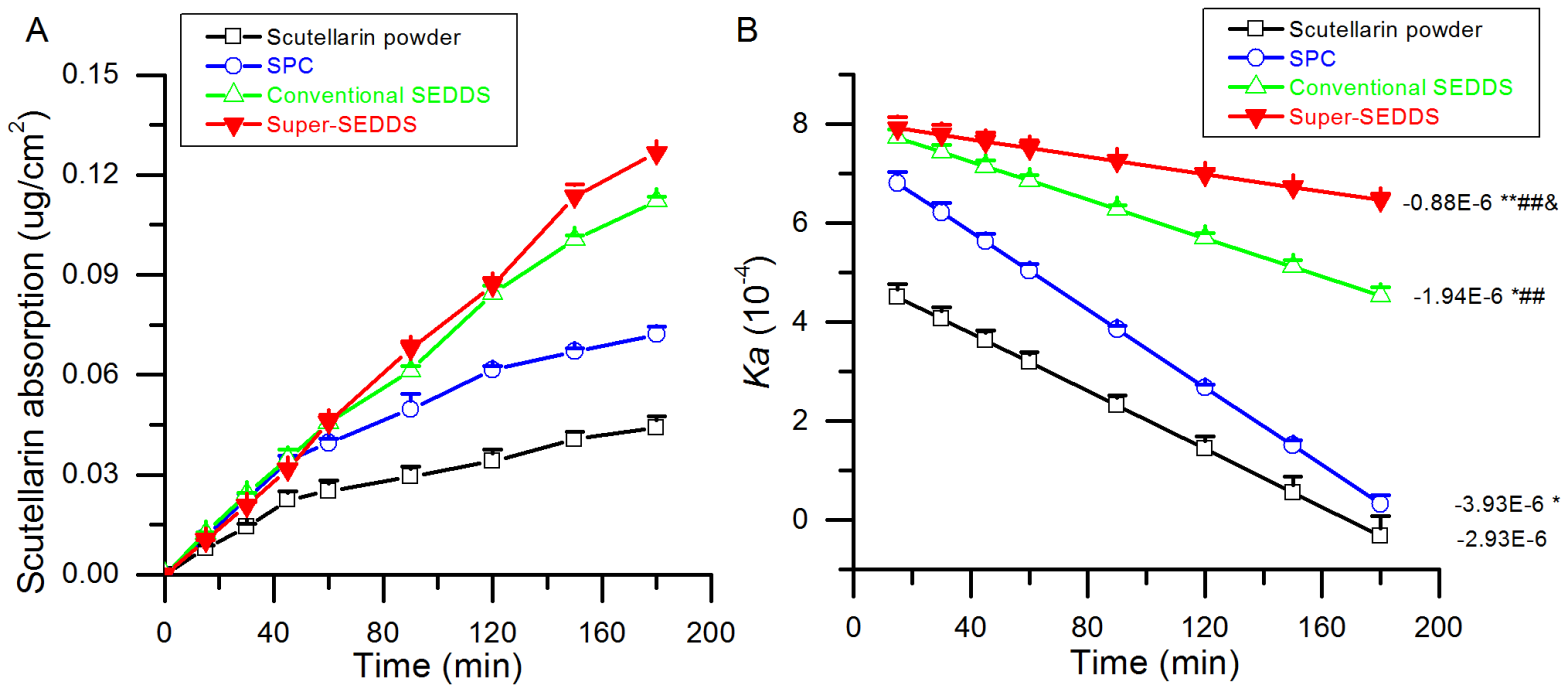
The intestinal absorption of scutellarin contained in various formulations. Cumulative absorption corresponding to various time points (A); Changes in *Ka* (absorption rate) over time (B), *p<0.05 and **p<0.001 compared with scutellarin powder, ^##^ *p*<0.001 compared with SPC, and &*p*<0.05 compared with conventional SEDDS (n = 3).

Many drugs on the market are faced with hindrances in their intestinal absorption rate, such as poor solubility, instability and unacceptable permeability on the variable conditions of the GI tract [30]. However, the intestinal absorption of some low solubility drugs has been successfully improved by the phospholipid complex method [19], [31], [32]. In the current study, the *ex vivo* intestinal absorption of scutellarin powder, SPC, conventional SEDDS and Super-SEDDS was investigated. The results correlated with those from the *in vitro* lipolysis study. As Figure 9A shows, the cumulative absorption of scutellarin, dosed in various formulations, indicated that SPC improved absorption compared with scutellarin powder. Both kinds of SEDDS showed further enhanced absorption. The results could be attributed to the self-aggregation of SPC as an amphipathic material to form micelles in aqueous media; an assumption which was outlined in previous research on salvianolic acid B phospholipid complex [31]. Furthermore, absorptions of scutellarin dosed in conventional SEDDS and Super-SEDDS were increased as a result of the emulsification of SEDDS. As can be seen in Figure 9B, Super-SEDDS was maintained at the highest absorption rate and showed the slowest decrease compared with all the other formulations, perhaps benefiting from looser tight junctions from high surfactant concentration [33]. The surfactant-based loosening of tight junction is one of the widely recognized mechanisms for enhancing the oral absorption of drug via SEDDS, in spite of bringing along some toxicity to epithelial cells. Fortunately, the current donor concentration of Cremophor RH40 is much lower than that reported toxic concentration (5 mg/mL) [29], and the surfactant-induced hurt is usually reversible [34] if surfactant concentration limited in the safety range.

In addition, it would be fairly expected to combine *in vitro* digestion model with *ex vivo* absorption model thus to synthetically evaluate the release behavior and permeation progress of drug. However, combining digestion and absorption models is still far from standardized and straightforward. Due to the *in vivo* absorption taking place in a much more complex and varying medium, the relevance of absorption tests is questionable; that is, both digestion and absorption models just reflect the influence of formulation performance on absorption, rather than help for a full understanding of the importance of metabolism, precipitation and re-dissolution during *in vivo* progress as mentioned later.

### *In Vivo* Study

**Figure 10:**
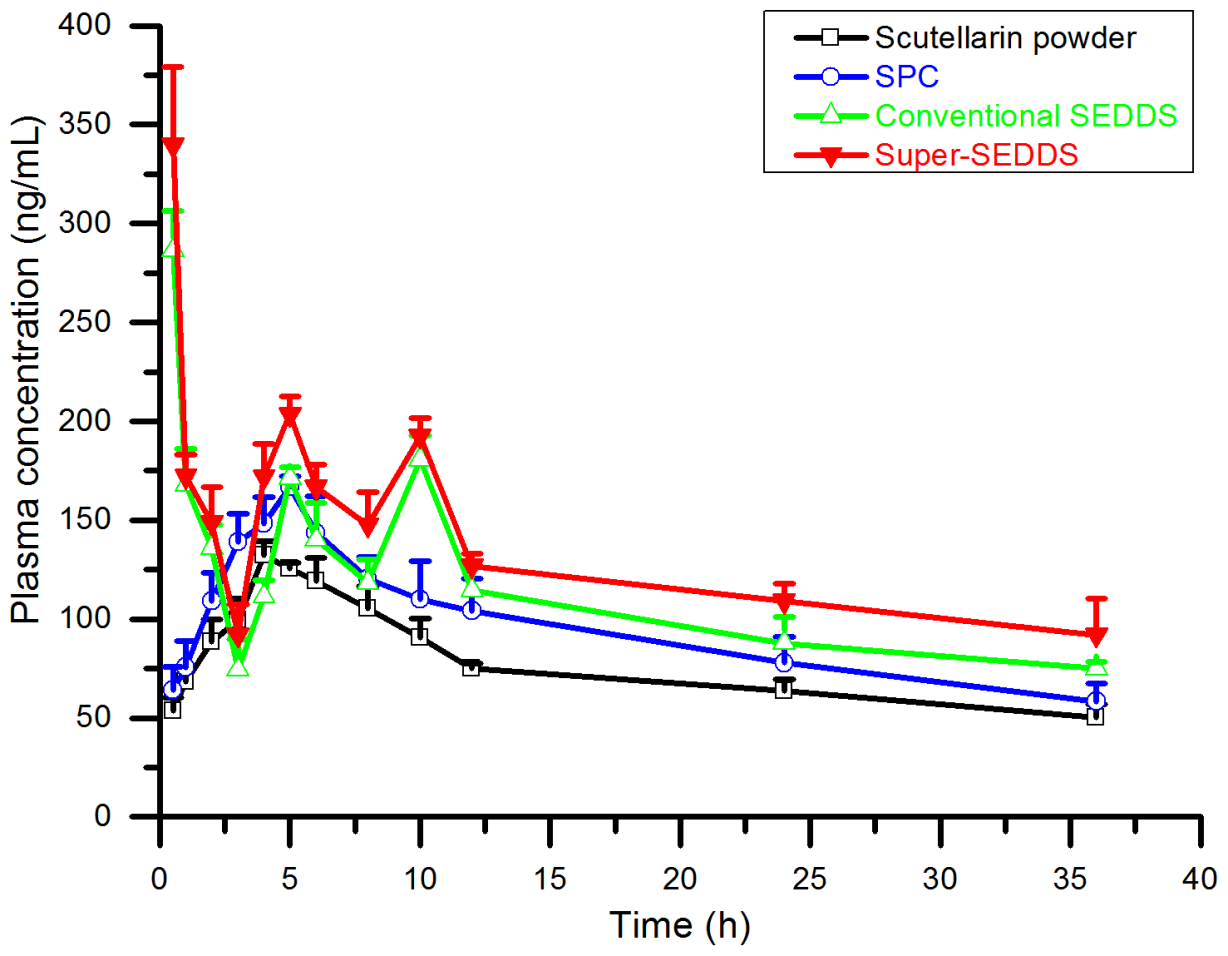
The plasma concentration-time curves of scutellarin. Scutellarin powder (black), SPC (blue), conventional SEDDS (green) and Super-SEDDS (red) were administrated to each group of SD rats (n = 5) respectively, with the dose of 40 mg/kg calculated in scutellarin. Blood samples were collected prior to the dose and at 0.5, 1, 2, 3, 4, 5, 6, 8, 10, 12, 24, and 36 h after the dose.

Although *in vitro* and *ex vivo* studies have proved the advantages of Super-SEDDS, *in vivo* pharmacokinetic study was still necessary for further demonstration. Figure 10 presents the mean plasma concentration versus time profiles of scutellarin, dosing with scutellarin powder, SPC, conventional SEDDS and Super-SEDDS respectively, while Table 5 summarized the corresponding pharmacokinetic data.

**Table 5:** Pharmacokinetic parameters of scutellarin orally dosed in various formulations (40 mg/kg equivalent to scutellarin) to SD rats (n = 5).

Formulations	C_max_ (ng/mL)	T_max_ (h)	T_1/2z_ (h)	AUC(0-t) (ng·h/mL)
Scutellarin powder	178±35	5.2±0.8	34.8±22.8	2673.5±404.1
SPC	198±41	4.2±0.8*	22.6±8.2	3374.9±573.2
Conventional SEDDS	290±39* #	0.6±0.2**^##^	25.9±9.2	4192.5±903.8*
Super-SEDDS	334±49**^##^	0.5**^##^	26.0±15.7	4612.4±631.1**#

Data represents mean ± SD, n = 5.

*p*<0.05 and **p<0.001 compared with scutellarin powder.

^#^ *p*<0.05 and ^##^ *p*<0.001 compared with SPC.

As Table 5 shows, the dosing of SPC provided no significant increases (*p*>0.05) in AUC_0-t_ and C_max_, compared with the dosing of scutellarin powder. Associating to the *in vitro* results, it can be concluded that *in vitro* advantages might not be inherited successfully *in vivo* for SPC administration. Until now, there have been few reports on the *in vivo* absorption of scutellarin dosed in SPC, even though it has previously been confirmed that the liposolubility and absorption of scutellarin in SPC has shown enhancements *in vitro* and *ex vivo* [19]. That could have resulted from degradation and hydrolysis by enzyme and bacteria when SPC was directly exposed in the GI tract [35]. Although scutellarin was modified as N,N-diethylglycollamide ester, similar challenges were continually revealed, until the drug was further encapsulated in emulsions, which could prevent degradation in the intestinal tract [36]. Inspired by this, SEDDS was proposed to compensate for the shortage of SPC.

Subsequent evidence certainly demonstrated that the absorption of scutellarin was improved by dosing in SEDDS. Compared with scutellarin powder, administration in both conventional and supersaturated SEDDS brought a significant (*p*<0.05) increase in AUC, approximately 1.6 to 1.7-fold. Even compared with SPC, the dosing of conventional SEDDS resulted in a slight increase in the oral absorption (*p*>0.05), while the use of Super-SEDDS showed a significant increase (*p*<0.05). These results were anticipated, as the emulsification of SEDDS facilitated the solubilization of SPC by forming mixed micelles, while the participation of phospholipid generated more negative charges for stabilizing emulsion droplets. The benefits of solubilization have also been highlighted in previous research, where some new formulations tried to improve the absorption of scutellarin. For example, β-cyclodextrin complex promoted absorption via passive diffusion and increased the water solubility 148-fold after encapsulating scutellarin through the hydrophilic and hydrophobic effect [37]. Similarly, the liposomal breviscapine increased C_max_ and AUC, which benefited from the excellent dissolution behavior of liposomal formulation [38]. It is also suggested that the formation of micelles from SEDDS may enhance the mass transport of molecules across the unstirred water layer in GI tract [39]. All these antecedents and recognitions have reiterated that SEDDS can be fully capable of promoting the dispersion of SPC in emulsified forms and improving the transportation of scutellarin. In addition, it should be concerned that the plasma concentration time profiles for conventional and super SEDDS are odd in shape, with high peak at a very early time point and multiple peaks following. The initial high peak may reflect the drug present in the intestine at supersaturated concentrations thus promoting rapid initial absorption, while the multiple peaks may reflect the following precipitation and re-dissolution events in the intestinal tract.

However, the clinical requirements for drug loading rate remained in danger of being unsatisfied if scutellarin was administrated in conventional SEDDS style. In recent years, Super-SEDDS has been suggested to overcome the oral absorption problems of poor water-soluble drugs [16], [40], [41]. As summarized from previous studies, Super-SEDDS has normally been achieved by formula optimization [40], [41] or craft amelioration [16]. In the current study, super-saturation was disparately achieved by dispersing excess SPC in the SEDDS pre-concentrate. This was achievable owing to the inherent compatibility of phospholipid and SEDDS. According to traditional concepts, a drug has to be in solution prior to absorption. However, the precipitation of scutellarin was inevitable after SEDDS was emulsified due to limitations in the drug-loading capability of emulsion. The overloading of a drug is therefore generally avoided, as the subsequent precipitates might adversely affect absorption. However, the results were seen to contradict this assumption. In fact, the amorphous precipitate originating from Super-SEDDS was proven to consistently contribute to absorption in the previous report [16], and in the current study, the SPC loaded Super-SEDDS proved advantageous in promoting intestinal absorption and *in vivo* bioavailability of scutellarin. The bioavailability of scutellarin dosing in Super-SEDDS was 4612.4 ng⋅h/mL, an almost 1.7-fold increase compared with dosing in scutellarin powder (2673.5 ng⋅h/mL). It was also slightly higher than that seen with dosing in conventional SEDDS (4192.5 ng⋅h/mL). In addition, we assume that the emulsifying of Super-SEDDS might form local supersaturated emulsions, which would benefit scutellarin avoiding conjugation with metabolic enzymes thus leaving more prototypal drug absorbed. Therefore, supersaturation does not need to be avoided, as enzyme-saturated absorption could potentially override the assumed negative effects of overloading. However, more details of this assumption need to be proved in future studies. In summary, SPC loaded Super-SEDDS, based on the strengths of SPC, SEDDS and supersatuation, have shown strong potential for use with high dosing drugs with poor water-solubility and meager liposolubility. As this is the first initial investigation of SPC loaded Super-SEDDS, many issues still remain and require further investigation, such as precipitation during *in vitro* release and the influence of precipitate on drug absorption *in vivo*.

## Conclusion

In the current study, a new strategy was provided to overcome the shortcomings of conventional SEDDS through the combination of phospholipid complex and supersaturated SEDDS. SPC was firstly prepared to enhance the liposolubility of scutellarin. Then SEDDS loading with SPC rather than scutellarin powder were designed to satisfy well dispersion of drug even at effective dose. Based on this, Super-SEDDS (200% S_eq_) as well as conventional SEDDS (50% S_eq_) were shown to promote the *in vitro* dissolution and *ex vivo* intestinal absorption of scutellarin. Moreover, *in vivo* results confirmed that Super-SEDDS demonstrated the best performance among all the formulations. In conclusion, SPC loaded Super-SEDDS was not just a simple combination of SPC and SEDDS, but also provided an advanced delivery system for compounds like scutellarin, with poor water-solubility, low liposolubility and high dose. As a potential alternate, this type of Super-SEDDS is anticipated to remedy the shortages of conventional SEDDS, in particular, high desire of lipophilic drugs and strict limitation of drug-loading rate.
