# Correction: A New Strategy for Enhancing the Oral Bioavailability of Drugs with Poor Water-Solubility and Low Liposolubility Based on Phospholipid Complex and Supersaturated SEDDS

**DOI:** 10.1371/journal.pone.0091605

**Published:** 2014-02-28

**Authors:** The PLOS ONE Staff

The legends for Figure 3 and 4 are swapped. Please see the corrected legends for Figure 3 and Figure 4 here.

**Figure 3:**
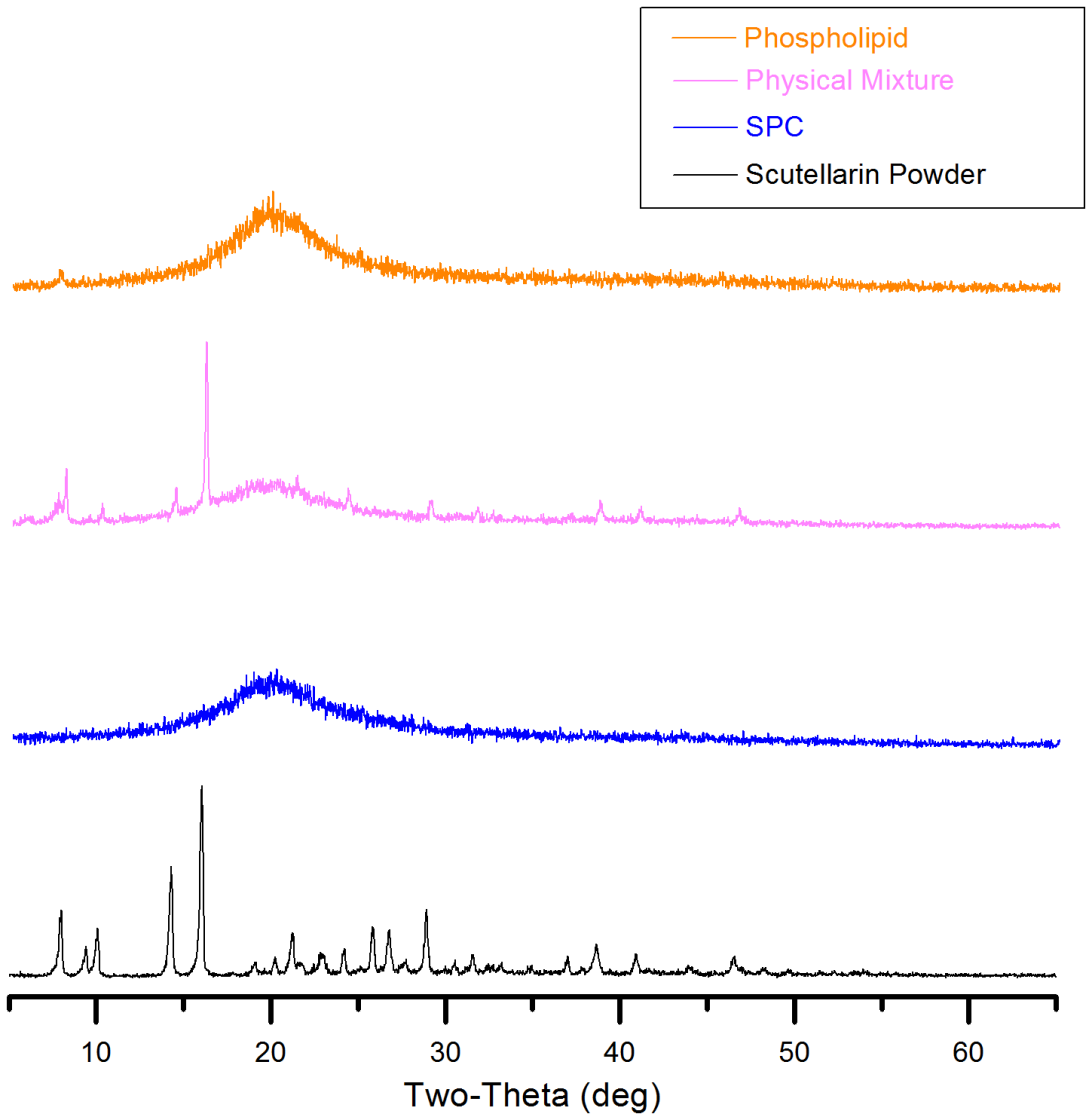
X-ray diffraction patterns of phospholipid, physical mixture, SPC and scutellarin powder. All samples were scanned over a range of 2 θ angles from 3° to 65° with an angular increment of 0.02° per second.

**Figure 4:**
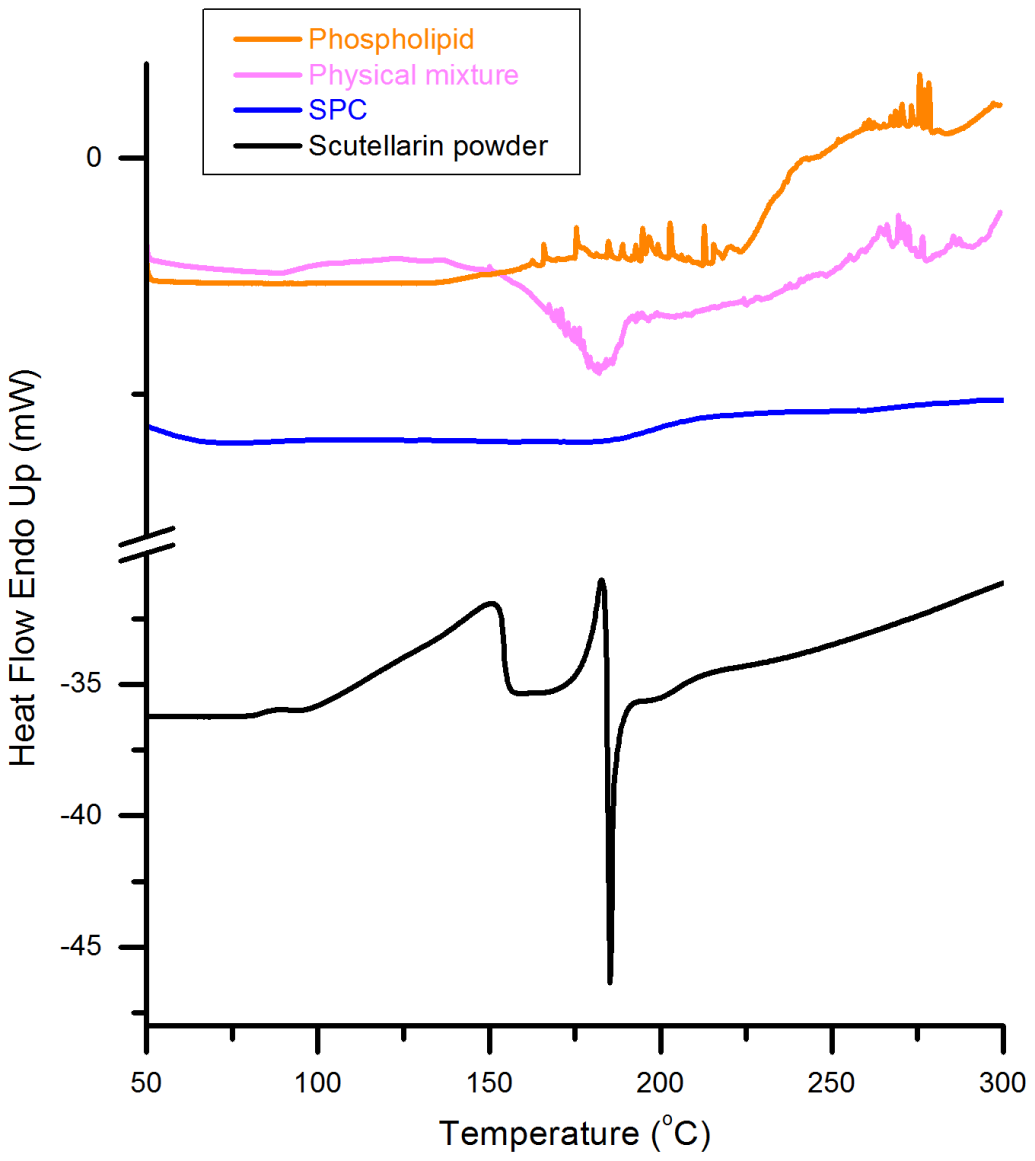
DSC thermograms of phospholipid, physical mixture, SPC and scutellarin powder . All samples were heated from 50 °C to 300 °C at a rate of 10 °C/min.
